# Thymoquinone Upregulates Catalase Gene Expression and Preserves the Structure of the Renal Cortex of Propylthiouracil-Induced Hypothyroid Rats

**DOI:** 10.1155/2020/3295831

**Published:** 2020-07-20

**Authors:** Nasra Ayuob, Maha Jameal Balgoon, Ahmed A. El-Mansy, Wafaa A. Mubarak, Alaa El-Din L. Firgany

**Affiliations:** ^1^Department of Histology, Faculty of Medicine, Delta University for Science and Technology, Gamasa City, Mansoura, Egypt; ^2^Yousef Abdullatif Jameel, Chair of Prophetic Medical Applications (YAJCPMA), Faculty of Medicine, King Abdulaziz University, Jeddah, Saudi Arabia; ^3^Department of Biochemistry, Faculty of Science, King Abdulaziz University, Saudi Arabia; ^4^Department of Histology and Cell Biology, Faculty of Medicine, Mansoura University, Egypt; ^5^Department of Histology, Hours University, Egypt; ^6^Department of Anatomy, Faculty of Medicine, Assiut University, Egypt; ^7^Department of Basic Medical Sciences, Unaizah College of Medicine and Medical Sciences, Qassim University, Saudi Arabia

## Abstract

**Background:**

The association between hypothyroidism and renal diseases has been described in many studies. *Nigella Sativa* was among the recently reported natural product that has the potential to prevent renal tissue damage and fibrosis. The aim of this study was to evaluate the possible protective effect of thymoquinone on the structure of the renal cortex of hypothyroid rats and explore the mechanism behind it.

**Methods:**

An experimental model of hypothyroidism was induced in adult male Wistar rats by administration of propylthiouracil (6 mg/kg/body weight). One hypothyroid group was treated with thymoquinone at the dose of 50 mg/kg/body weight and compared to the untreated group. Thyroid function and oxidant/antioxidant status were assessed in the serum. Catalase gene expression was assessed using the real-time polymerase chain reaction. The kidney was assessed both histologically and immunohistochemically.

**Results:**

Administration of propylthiouracil resulted in a significant decrease in the serum levels of nitric oxide, reduced glutathione, and superoxide dismutase activity while the level of malondialdehyde significantly (*p* < 0.001) increased. Administration of thymoquinone alleviated this effect on the thyroid hormones and significantly increased the serum levels of antioxidants. Thymoquinone significantly (*p* < 0.001) upregulated catalase transcription by about 24-fold and could block the hypothyroidism-induced glomerular and tubular injury.

**Conclusion:**

Thymoquinone may have a potential protective effect against hypothyroidism-induced renal injury acting through the attenuation of the oxidative stress and upregulation of renal catalase gene expression.

## 1. Introduction

Although diseases of thyroid dysfunction are common, readily identifiable, and treatable conditions, they could result in severe adverse effects if undiagnosed or untreated [1].

The association between hypothyroidism and renal diseases has been described in many studies [2]. Kalashnikova et al. had reported that thyroid hormone deficiency was associated with reduced renal blood flow, impairment of renal filtration, tubular reabsorption, and secretory functions [3]. High TSH levels are positively correlated with the prevalence of chronic kidney disease (CKD) and are considered a risk factor for CKD development [4]. An increase in the level of creatinine in the serum of hypothyroid patients has been recently reported.

On the other hand, thyroid dysfunction has been proposed to occur in patients with CKD [5]. In a recent study conducted in 2020 by Nazzal et al. in Palestine, it was reported that the prevalence of overt (9.1%) and subclinical (7.2%) hypothyroidism becomes common in patients on renal dialysis. They recommend conducting screening programs and more studies on the efficacy of treatment of such condition [6]. Severe cases of hypothyroidism can lead to renal failure. However, if hypothyroidism is early recognized and treated, rapid and complete resolution of severe renal impairment can be achieved [7].

Many natural products were reported to have a protective effect against many diseases due to their antioxidant and anti-inflammatory effects [8]. Thymoquinone (TQ) is a predominant, potent, and pharmacologically bioactive constituent of the annual herbaceous plant *Nigella sativa L* (black seed) that has been commonly used as a natural remedy for various diseases for over 2000 years. Among the various therapeutic potentials and activities that have been attributed to TQ after being widely studied were the antibacterial, anticancer, anti-inflammatory, and antioxidant effects [9].

Previous studies conducted on *Nigella sativa* (NS) oil and its main bioactive constituent TQ showed that they have both antioxidant and renoprotective properties in STZ-induced diabetes in the animal models and in ischemia-reperfusion-induced renal disorders [10, 11]. In addition, NS and TQ were recently reported to have the potential to prevent renal tissue damage and fibrosis in lipopolysaccharide- (LPS-) treated rats through its antioxidant and anti-inflammatory effects [12, 13]. It was reported that the hydroalcoholic extract of NS could protect the renal tissue against oxidative stress associated with neonatal and juvenile hypothyroidism in rats [14]

None of the available studies neither investigated the effect of the TQ in specific, the bioactive constituent of NS, on the structure of the renal tubules of hypothyroid animal model nor tried to explore the mechanism behind this effect. Therefore, the aim of this study was to evaluate the possible protective effects induced by TQ on the histological and biochemical changes that occur in the renal cortex of hypothyroid rats and elucidate the mechanism by which these effects were induced.

## 2. Materials and Methods

This study design was reviewed and permitted by the Biomedical Research Ethics Committee at the Faculty of Medicine, King Abdul Aziz University (KAU), Jeddah, Saudi Arabia. The experiment was conducted at King Fahed Medical Research Center (KFMRC) at KAU in collaboration with the research center in the Mansoura Faculty of Medicine with full consideration of the guidelines of animal care set in these research centers.

### 2.1. Chemicals

Thymoquinone extracted from *N. sativa* was purchased from Frinton Laboratories Inc. and dissolved in dimethyl sulfoxide (DMSO) (at the dilution 1 : 100). TQ was given at the dose of 50 mg/kg/body weight/daily through IGI [15]. Propylthiouracil (PTU) was purchased from Sigma-Aldrich Inc. (St. Louis, Missouri, Sylon, Boulevard, Hainesport, USA). PTU was given through IGI (6 mg/kg/body weight/daily) for 6 weeks as was described by Villar et al. [16]

Levels of T3 (Bayer ADVIA ® Centaur*™* FT_3_, Bayer Corporation, Norwood, MA), T4 (Bayer ADVIA ® Centaur*™* FT_4_, Bayer Corporation, Norwood, MA), and TSH (Bayer ADVIA ® Centaur*™* TSH, Bayer Corporation, Norwood, MA) in the serum were measured using ADVIA Centaur automated competitive chemiluminescence immunoassay Bayer HealthCare (Kanawha River, Western West Virginia, USA). Levels of malondialdehyde (MDA) (Biodiagnostic, Giza, Egypt, CAT. No. 2529), reduced glutathione (GSH) (Biodiagnostic, Giza, Egypt, CAT. No. 2511), and nitric oxide (NO) (Biodiagnostic, Giza, Egypt, CAT. No. 2533). Glutathione peroxidase (GPX) (Biodiagnostic, Giza, Egypt, CAT. No. 2524), superoxide dismutase (SOD) (Biodiagnostic, Giza, Egypt, CAT. No. 2521), and catalase activities (Biodiagnostic, Giza, Egypt, CAT. No. 2517) were assessed.

### 2.2. Experimental Design

Twenty-four adult male Wister rats (180–200 g) purchased from the animal house at KFMRC were kept to acclimatize for 2 weeks in the standard laboratory conditions. The rats were randomly divided into four groups (*n* = 6). The whole experiment continued for 6 weeks. During this period, the rats were maintained on commercial food ad libitum consisting of standard laboratory rat chow (Grain Silos and Flour mills organizational laboratory animal, F-648 net weight 50 kg).

Rats of the negative control group received 1 ml of DMSO through intragastric intubation (Arab, #107) for 6 weeks. The positive control group (TQ-treated) was given TQ through IGI for 6 weeks. Hypothyroidism was induced in the other two groups by the administration of PTU through IGI for 6 weeks. After two weeks of PTU administration, the hypothyroid state was confirmed in the rats by assessing the T3, T4, and TSH levels in the serum. T3 level which is equal to 107.2 ± 0.8 (ng/ml) or less, T4 level which is equal to 1.7 ± 0.6 (ng/ml) or less, and TSH (mIU/l) level which is equal to 19.60 ± 3.08 or more were considered confirmatory of hypothyroidism [17]. The hypothyroid rats were then divided into two groups: the hypothyroid group continued on PTU alone without treatment for another 4 weeks and the other hypothyroid group treated with TQ (hypothyroid+TQ) for 4 weeks.

### 2.3. Biochemical Techniques

Blood samples were obtained for biochemical assessment from the intraorbital sinus during the experiment and from the heart at the end of the experiment [18]. Plasma was obtained from part of the sample. Centrifugation at 3000 rpm for 15 min at 4°C was performed to obtain the serum from part of the blood samples and was kept at −18°C. Levels of T3, T4, and TSH were assessed in the serum while MDA, GSH, NO, GPX, SOD, and catalase were assessed in the plasma.

### 2.4. Gene Expression of Catalase

The RT-PCR assay was performed on Real Time PCR system (Applied Biosystems, 7500, USA) and 2X SYBR Green PCR Master Mix (Applied Biosystems, USA). The primer sequences for catalase gene (GenBank accession no. NM_012520) were forward GAATGGCTATGGCTCACACA and backward CAAGTTTTTGATGCCCTGGT. The housekeeping gene—GAPDH—was used as a control for normalization with primer sequence forward ATGGAGAAGGCTGGGGCTCACCT and backward AGCCCTTCCACGATGCCAAAGTTGT.

Optimization of the primer concentration was performed to determine the primer concentration that gives the lowest threshold cycle (CT). This optimization was performed by the creation of a matrix of reactions to test a range of concentrations for each primer against different concentrations of the partner primer. A NanoDrop 2000 Spectrophotometer (Thermo Fisher Scientific, USA) was used to ensure the purity of the extracted RNA and minimize the nonspecific amplification in real-time RT-PCR reactions.

The assay was performed as defined by Bunker et al. [19]. In a 25 *μ*l reaction combination consisting of 12.5 *μ*l 2X SYBR green PCR master mix, 2 *μ*l cDNA, and 10 pmol of both forward and reverse primers. After optimization, the thermal cycling was performed as it adheres to the following: initial hold at 50° C for 2 minutes, initial denaturation at 95° C for 10 minutes complied with 40 amplification cycles (95° C 15 s and also 60° C for 1 minutes), and ultimately an added action (60° C for 15 s, 95° C for 15 s, and 37° C for 2 minutes). The PCR response was duplicated in 3 in six animals.

**Figure 1:**
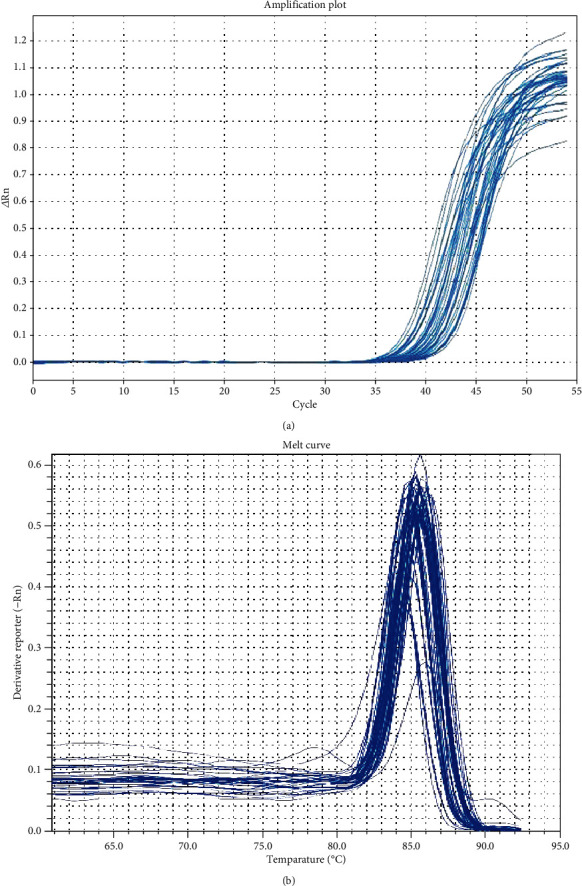
Catalase gene real-time PCR amplification plot (a) and melting curve (b).

The PCR reactions were kept track by determining the strength of the fluorescence brought on by SYBR Green Dye intercalation to the ds DNA; melting curve evaluation was done to verify the specificity of the products (Figures 1(a) and 1(b)). The CT values were figured out after that relative quantification using the *Δ*CT method with reference gene. The calculation differences between GAPDH (endogenous reference gene) and catalase CT value for every sample were computed; then, the ratio of relative expression was determined.

### 2.5. Histological Techniques

At the end of the 6^th^ week, rats were lightly anesthetized using diethyl ether then decapitated. The chest wall was opened, and the blood was obtained rapidly from the heart. The kidney was immediately and gently dissected out and fixed in 10% neutral buffered formalin to be further processed for obtaining paraffin blocks. Paraffin sections at 4 *μ*m thickness were prepared and stained with hematoxylin and eosin. Other sections were stained with Masson's trichrome stain for the demonstration of collagenous fibers [20].

In addition, another set of paraffin sections at same thickness was immunohistochemically stained using the streptavidin–biotin–peroxidase technique described by Gu and Herrera [21]. Anti-alpha-smooth muscle actin (ASMA) antibody (Dako Company, Cairo, Egypt; at a dilution of 1/1000), antiendothelial nitric oxide synthetase (eNOS) antibody (Dako Company, Cairo, Egypt; at a dilution of 1/100), and anti-CD68 antibodies were utilized in this study. The primary antibody was omitted during staining of some slides to be used as a negative control. The nuclei were counterstained with hematoxylin. Brown cytoplasmic staining was considered positive reaction in ASMA, CD68, and eNOS antibodies.

Olympus Microscope BX-51 (Olympus) connected to a digital camera and a computer was used for photographing. Pro Plus image analysis software (version 6.0) (Media Cybernetics, Inc. Rockville, MD 20850 USA) was used for semiquantitative analysis of antibody immunoreactivity. The area percentage of immune-positive cells stained with ASMA, eNOS, and CD68 antibodies, as well as Masson-stained collagen fibers, was assessed in 30 fields using a ×40 objective lens and ×10 ocular lens [22]. The diameter of the renal glomeruli was measured by using ×40 objective lens and ×10 ocular lenses. Ten randomly selected different glomeruli in the peripheral cortex of each section were evaluated. The diameters of the greatest and the smallest glomerulus were measured, and the average diameter was calculated and the mean was calculated for each animal.

### 2.6. Statistical Assessment

The data of this study were analyzed using the statistical package for the social sciences (SPSS, version 16; SPSS Inc., Chicago, Illinois, USA). The results were expressed as mean ± standard deviation SD. The normality of the distribution of the data was assessed using the Kolmogorov–Smirnov test because it works well with samples of identical values which happened sometimes in the studied variables. Regarding the parametric data, the different groups were compared using the analysis of variance (ANOVA) then the Bonferroni post hoc test to avoid a multiple comparison effect. For nonparametric data, the Kruskal–Wallis test and then Dunn procedure post hoc test were used. Statistical significance was accepted at *p* value less than <0.05.

## 3. Results

### 3.1. Effect of TQ on Serum Level of T3, T4, and TSH

**Figure 2:**
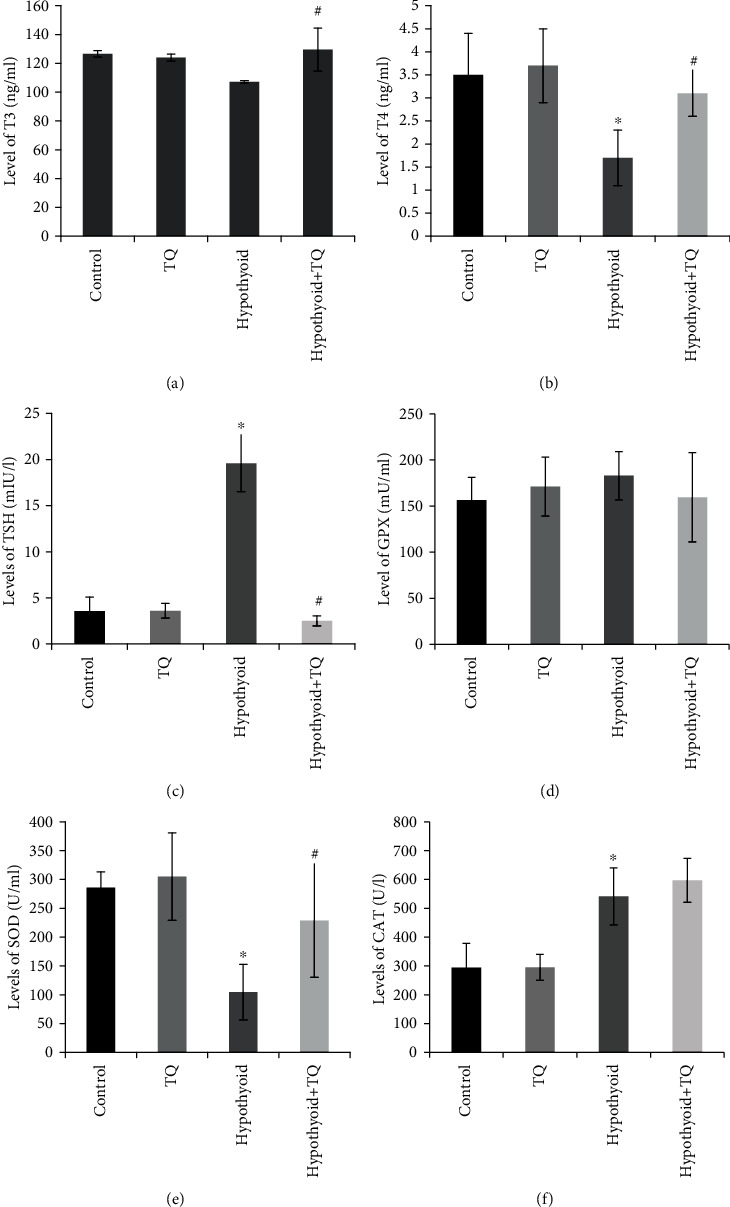
Effect of TQ on serum level of thyroid hormones (a, b), TSH (c), and enzymatic antioxidant activities included GPX (d), SOD (e), and CAT (f).

A significant reduction of the T4 (*p* = 0.002) levels was observed in rats that received PTU for 6 weeks compared to control rats, while T3 showed insignificant reduction. On the other hand, the TSH level was significantly increased (*p* < 0.001) in rats that received PTU compared to the control rats. It was found that administration of TQ induced a significant increase in the levels of T3 (*p* = 0.004) and T4 (*p* = 0.001) as well as a significant decrease in the level of TSH (*p* < 0.001) compared to those of the hypothyroid group (Figures 2(a)–2(c)).

### 3.2. Effect of TQ on Plasma MDA, NO, and GSH in the Studied Groups

In the rats that received PTU for 6 weeks, the levels of NO and MDA significantly elevated (*p* < 0.001) while the GSH level showed a nonsignificant reduction compared to those of the control rat. Levels of NO and GSH significantly increased while the level of MDA significantly decreased in the hypothyroid+TQ group (Table 1).

### 3.3. Effect of TQ on Enzymatic Antioxidant Activities

The hypothyroid group showed a significant (*p* < 0.001) reduction in SOD activity while this activity significantly elevated (*p* = 0.04) in rats that received TQ. On the other hand, CAT activity significantly elevated (*p* < 0.001) in the hypothyroid group, and it showed a further nonsignificant increase in the hypothyroid+TQ group. The level of GPX did not show any significant changes in any of the studied groups (Figures 2(d)–2(f)).

### 3.4. Effect of TQ on Gene Expression of Catalase

The level of the renal catalase transcription showed a nonsignificant (*p* = 0.79) increase in rats in the TQ-treated group compared to the control group. Although catalase transcription was downregulated in hypothyroid rats, its mean expression showed a nonsignificant decrease. On the other hand, catalase transcription showed a significant upregulation (*p* < 0.001) by about 24-fold in the hypothyroid+TQ group compared to the control group (Table 1).

### 3.5. Renal Histopathological Changes

**Figure 3:**
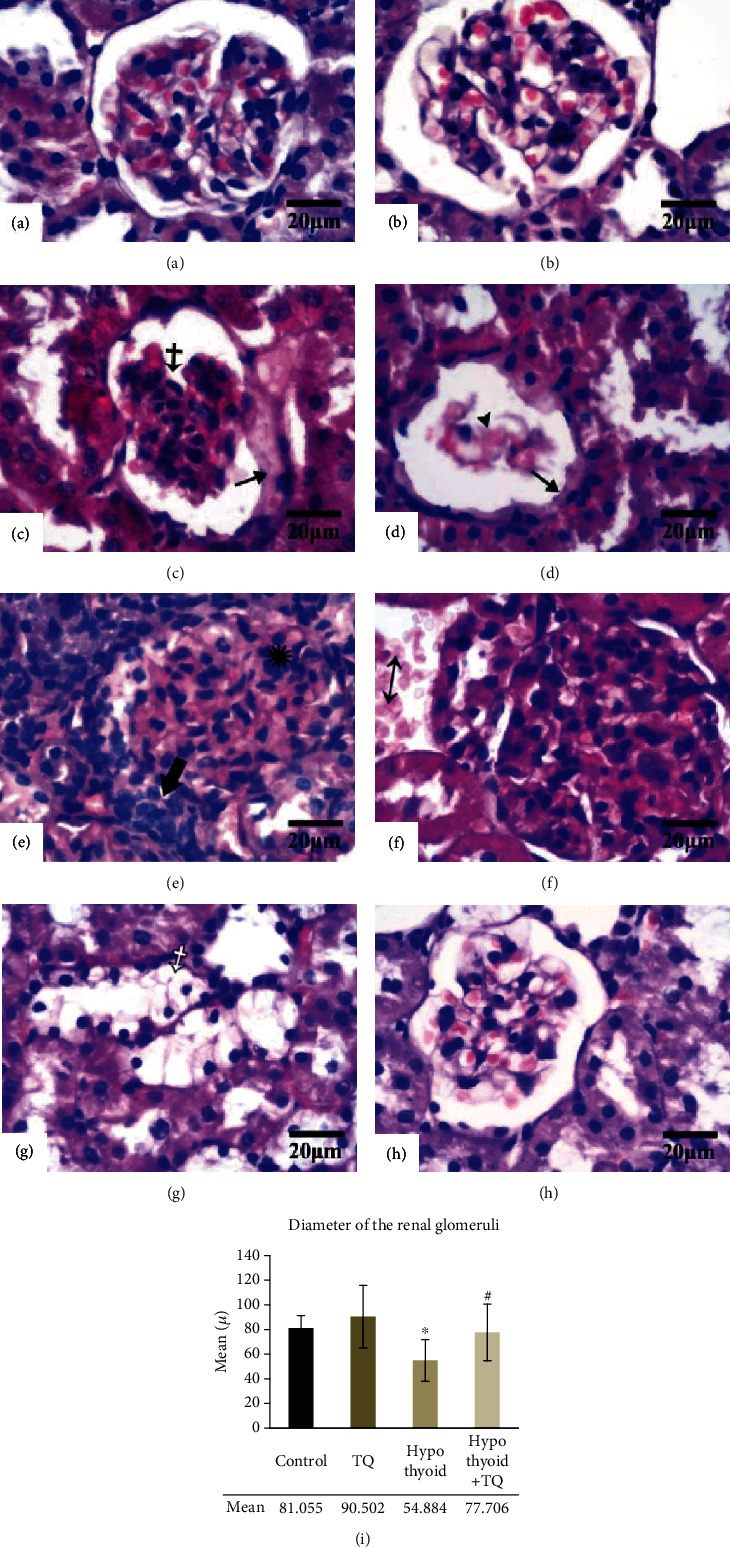
The renal cortex of the control (a), TQ (b), hypothyroid (c, d, e, f, g), and hypothyroid+TQ (h) groups. Renal corpuscles in the control and TQ groups appear normal while in the hypothyroid group some glomeruli show distortion (crossed arrow), atrophy (arrowhead) or mesangial proliferation (asterisk). The capsular epithelium appears swollen (thin arrows). Mononuclear cell infiltrate is seen in the capsular space of some glomeruli (thick arrow). The interstitium displays mononuclear cell infiltrate (empty arrow), proliferating fibroblasts (empty asterisk), hemorrhage (double head arrow). Some renal tubules exhibit clear cytoplasm (empty crossed arrow). The renal corpuscles of hypothyroid+TQ show mild distortion and renal tubules appear normal. (H&E, ×400, scale bar = 20 *μ*m). The diameter of the renal glomeruli (i) of the studied groups is represented.

As shown in Figure 3, the examination of H&E-stained renal cortical tissue of the control and TQ groups revealed preserved kidney architecture without detection of any tissue damage. Induction of hypothyroidism resulted in obvious histopathological changes. Some glomeruli were distorted or atrophied, and others displayed mesangial cell proliferation. The capsular epithelium appeared swollen (Figures 3(c)–3(e)). Mononuclear cell infiltrate was detected in the capsular space of some glomeruli and in the interstitium that showed also proliferating fibroblasts and hemorrhage (Figures 3(e)–3(h)). Nevertheless, the hypothyroid+TQ group showed that the glomerular and tubular injury was reversed. The renal corpuscles were slightly distorted, and most of renal tubules appeared normal (Figure 3(i)). The mean diameter of the renal glomeruli was significantly decreased (*p* < 0.001) in hypothyroid rats compared to the control while it was significantly increased (*p* = 0.02) in the hypothyroid+TQ group.

**Figure 4:**
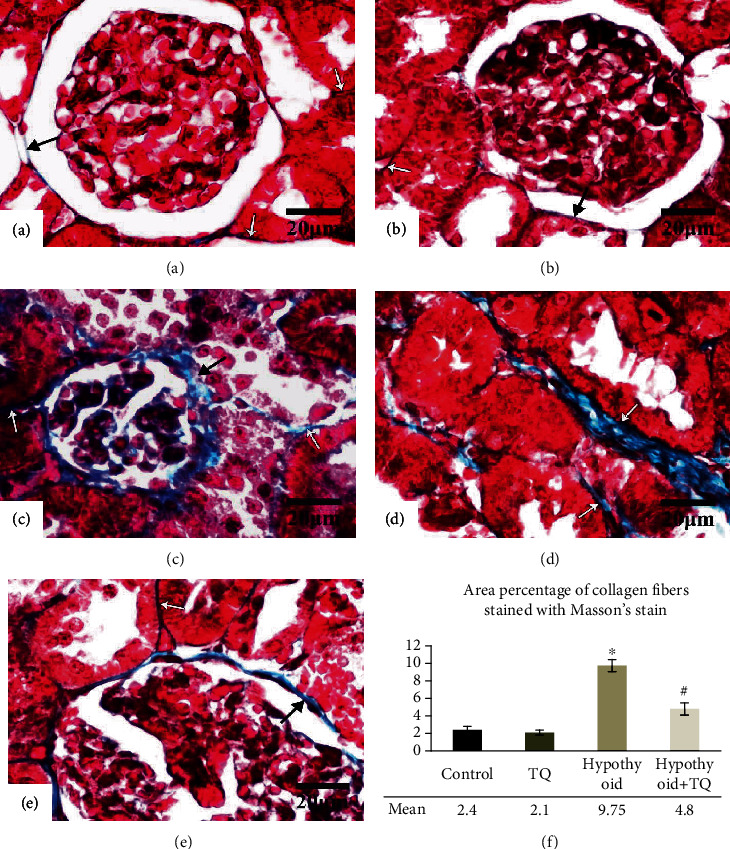
The renal cortex of the control (a), TQ (b), hypothyroid (c, d), and hypothyroid+TQ (e) groups. Little collagenous fibers are seen surrounding the renal corpuscle (thin arrow) and in the interstitium between the tubules (empty arrows) in the control and TQ groups. In the hypothyroid group, deposition of more collagenous fibers is marked around the renal corpuscle and in between the tubules. Less deposited fibers are noticed in the hypothyroid+TQ group (Masson's trichrome stain ×400, scale bar = 20 *μ*m). The area percent of Masson-stained collagen fibers (f) of the studied groups is represented.

Masson's trichrome-stained sections revealed that the amount of collagenous fibers around the renal corpuscles and in between the renal tubules was minimal in both the control and TQ groups. Marked deposition of more collagenous fibers was observed in the hypothyroid group while less marked deposition was noticed in the hypothyroid+TQ group (Figure 4). Semiquantitative assessment revealed a significant increase (*p* < 0.001) in the area percent of Masson-stained collagen fibers in the hypothyroid group compared to the control while it significantly decreased (*p* < 0.001) in the hypothyroid+TQ group.

### 3.6. Renal Immunohistochemical Changes

**Figure 5:**
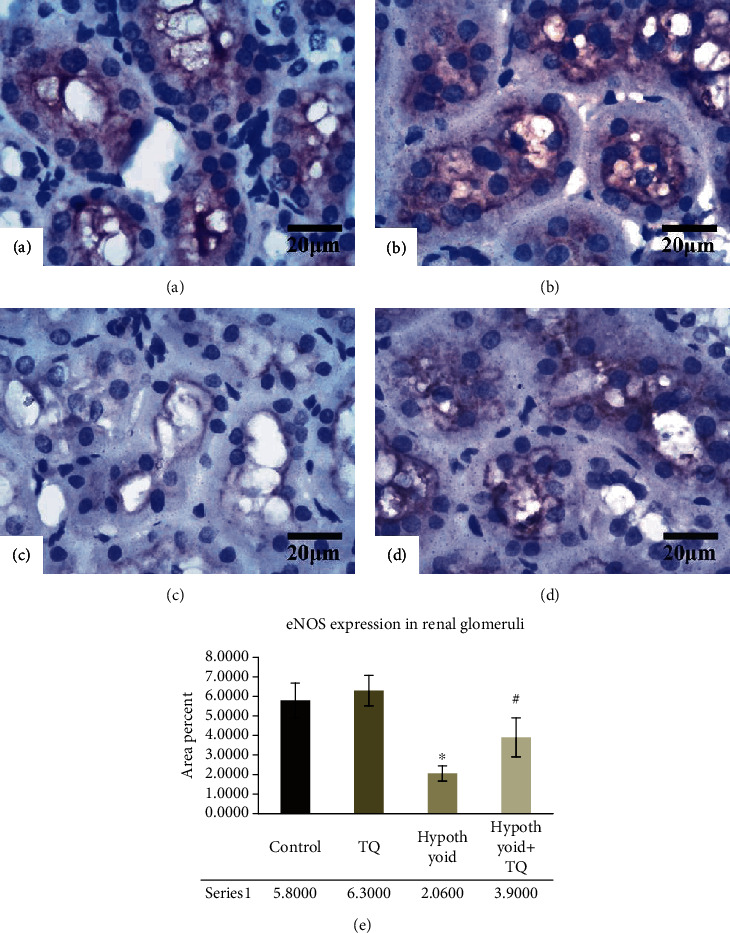
The cortical renal tubules exhibit a strong widely distributed eNOS cytoplasmic reaction in both the control (a) and TQ (b) groups. The hypothyroid group shows a weaker and more localized reaction (c). Hypothyroid rats administered TQ display more evident reaction in the renal tubules (d) (eNOS, ×400, scale bar = 20 *μ*m). eNOS expression in the renal glomeruli (e) of the studied groups is represented.

It was noticed that in the control group, eNOS was intensely expressed in wide cytoplasmic areas. The TQ group displayed a similar pattern of eNOS expression (Figure 5). On the other hand, the intensity of eNOS-positive reaction was significantly reduced (*p* < 0.001) in the hypothyroid group. However, hypothyroid+TQ showed a significant increase (*p* < 0.001) in the cytoplasmic eNOS expression in the tubular epithelium (Figure 5(e)).

**Figure 6:**
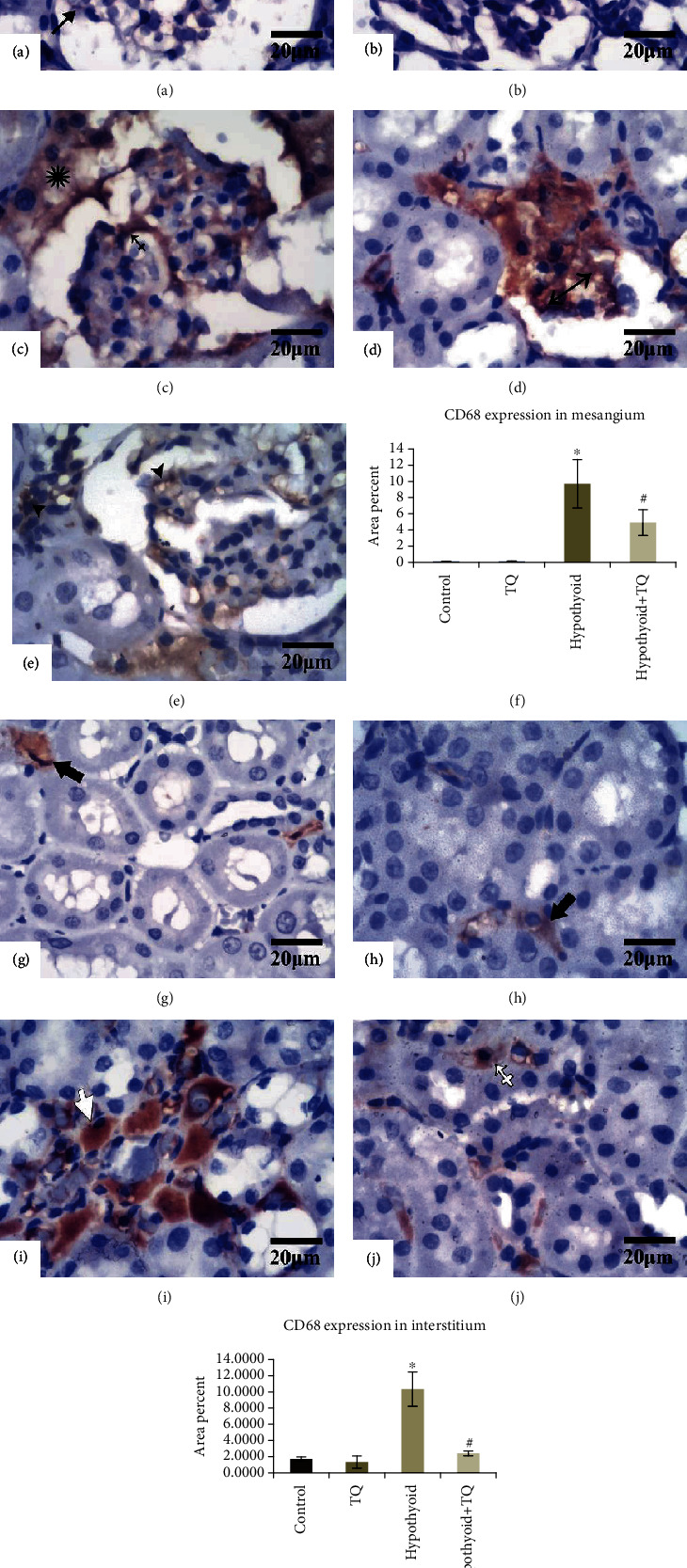
The glomeruli exhibit a very localized faint CD68 reaction (arrows) in both the control (a) and TQ (b) groups. The glomeruli in the hypothyroid group shows a strong widely distributed reaction (crossed arrow) with a periglomerular strong reaction (asterisk) (c). Some glomeruli are atrophied and totally replaced by CD68+ve cells (double headed arrow) (d). Hypothyroid rats administered TQ display a weaker and more localized glomerular and periglomerular reaction (arrowheads) in comparison with the hypothyroid group (e). The interstitium of both the control (f) and TQ (g) groups shows few CD68+ve cells (thick arrows). The hypothyroid group exhibit obvious increased the number of CD68+ve cells (empty arrow) (h). The hypothyroid+TQ group displays markedly decreased CD68+ve reaction (empty crossed arrow) (i) (CD68, ×400, scale bar = 20 *μ*m). CD68 expression in the renal mesangium (f) and interstitium (k) of the studied groups is represented.

As illustrated in Figure 6, the mesangium and the interstitium of both the control and TQ-treated groups showed a localized weak CD68-positive reaction. However, the hypothyroid rats developed an intense widely distributed and significant (*p* < 0.001) CD68-positive reaction in the glomeruli and in the periglomerular area compared to that of the control group. Some glomeruli were atrophied and entirely replaced by CD68-positive cells. However, the glomerular, periglomerular macrophage, and interstitial macrophage infiltration was significantly reduced (*p* < 0.001) in the hypothyroid+TQ group (Figures 6(f) and 6(k)).

**Figure 7:**
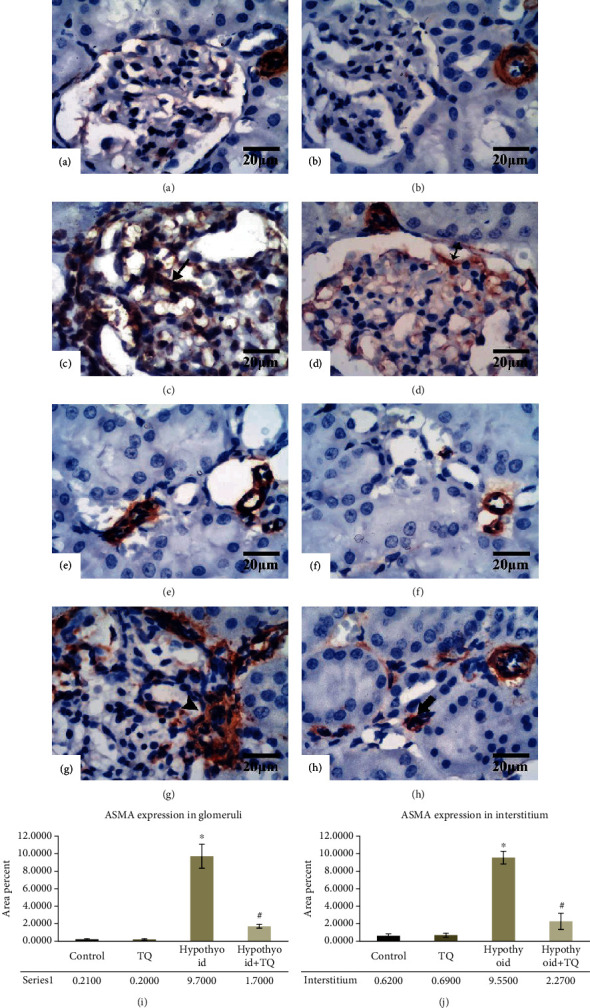
The glomeruli exhibit a -ve ASMA reaction in both the control (a) and TQ (b) groups. The glomeruli in the hypothyroid group show a strong widely distributed reaction (Shah, #102) (c). Hypothyroid rats administered TQ display a weaker and more localized glomerular reaction (crossed arrow) (d). The interstitium of both the control (e) and TQ (f) groups exhibits no ASMA+ve cells. The hypothyroid group reveals abundant ASMA+ve cells (arrowhead) (g). The hypothyroid+TQ group displays a fewer number of ASMA+ve cells (thick arrow) (i) (ASMA, ×400, scale bar = 20 *μ*m). ASMA expression in the renal glomeruli (i) and interstitium (j) of the studied groups is represented.

Regarding ASMA, Malpighian corpuscles as well as the renal cortical interstitium of both the control and TQ-treated groups showed negative reaction (Figure 7). Nevertheless, the glomeruli in the hypothyroid group exhibited a widely distributed strong ASMA-positive reaction that was significantly higher (*p* < 0.001) than that in the control group. On the other hand, hypothyroid rats had a significant reduction (*p* < 0.001) in the intensity and a retraction of the area of ASMA-positive reaction in the glomeruli of the hypothyroid+TQ group (Figures 7(i) and 7(j)). In addition, the peritubular interstitium of the hypothyroid group showed numerous ASMA-positive cells. However, the number of ASMA-positive detected cells was obviously diminished in the hypothyroid+TQ group in comparison to the hypothyroid group.

## 4. Discussion

Hypothyroidism is one of the most common causes of distal renal tubular acidosis [23]. The aim of this study was to investigate the effects of administration of TQ on the structure of the kidney of hypothyroid rats. The TQ-induced protective effect against tissue oxidative damage was also investigated as a possible mechanism underlying such effect. In the present work, renal cortical tissue obtained from hypothyroid rats showed obvious histopathological changes including glomerular distortion, atrophy, and intracapsular mononuclear cell infiltrate with proliferation of mesangial cells and swelling of the capsular epithelium. These changes represented components of the various forms of glomerulonephritis previously reported by Singh et al. [2] in renal biopsies from hypothyroid patients and by Wang and Pei [24] in hypothyroid rats. The mechanism of such injury is multifactorial and may include changes in systemic and intrarenal hemodynamics, direct effects on renal tubular function, and hypothyroidism-associated rhabdomyolysis [6]. In this study, after the hypothyroid rats received TQ, the glomerular and tubular injury was nearly improved except for slight distortion of renal corpuscles. In the same context, treatment with TQ was reported to preserve the glomerular structure after renal injury induced by the administration of the chemotherapeutic agent doxorubicin [9].

The biochemical results of T3, the biologically active thyroid hormone, T4, and TSH showed that PTU exposure resulted in hypothyroidism. However, TQ administration improved the disturbed thyroid function. In the same context, Baghcheghi et al. reported that thymoquinone significantly increased the T4 level in PTU-induced juvenile hypothyroid rats [25]. In addition, it was reported that antioxidant agents, such as vitamin C, improved the disturbed serum T4, T3, and TSH levels in patients with hypothyroidism [26]. Therefore, increasing the level of thyroid hormones could be a possible mechanism that indirectly contributed to the improving effects induced by TQ on the renal structure in the current experiment.

In the present study, eNOS was intensely expressed in wide cytoplasmic areas in the cortical renal tubules of the control group while it is markedly suppressed by induction of hypothyroidism. Rodríguez-Gómez et al. [27] stated that the eNOS level dropped not only in renal tissue but also in the heart and aorta of hypothyroid rats. A variety of factors is involved, either alone or in combination, in the modulation of expression of eNOS in hypothyroid rats. These factors include the direct impact of thyroxin on eNOS activity, changes in blood pressure, altered levels of vasoactive agents, and/or changes in shear stress due to hypodynamic circulation of hypothyroid rats [26]. Conversely, TQ administration increased eNOS expression in tubular epithelium possibly through its antioxidant properties. In spite of impairment of eNOS expression in renal tubules and deterioration of redox status serum markers in the hypothyroid group, the same animal group recorded a significant rise in the serum level of NO probably as a protective mechanism against oxidative stress exerted by thyroxin deficiency.

It was reported that hypothyroidism is associated with oxidative stress status [28]. On analyzing biochemical results of this study, it was noticed that the animals that received PTU showed deterioration of the antioxidant parameters in the form of significant elevation of MDA serum level and drop of both SOD and GSH levels. In line with these results, we found that the level of renal catalase transcription was downregulated in hypothyroid rats. These data are in agreement with previous reports that showed decreased SOD and CAT activities and increased MDA level in both renal tissue and serum of hypothyroid rats [5, 29]. Considering these results, tissue oxidative damage as a possible mechanism for deleterious effects of hypothyroidism on renal tissue architecture might be suggested.

The effect of PTU on catalase gene expression was contradictory; we observed a decrease in the activity of catalase gene in PTU-induced hypothyroid rats. Our finding was in agreement with that of Bunker et al. [19] who reported a significant decrease in catalase gene expression by fivefold in rats treated with PTU for 90 days. On the other hand, our finding is against the published work of Chattopadhyay et al., who reported an increased activity of CAT in the heart of hypothyroid rat which was suggested to be a reflex mechanism against increased oxidative stress induced by hypothyroidism [30]. On the other hand, the current study demonstrated a significant upregulation of catalase gene expression in the kidney of the hypothyroid+TQ group, which was in an agreement with Khan et al. [31]. Mohebbati et al. [14] also reported an increase in CAT and SOD activity in renal tissues following administration of 200 and 400 mg/kg of *N. sativa* extract compared to the PTU group.

The scavenging power of TQ was described to be augmented by many factors including “the redox properties of its quinone structure, its unlimited ability to cross the biological barriers and subsequent easy access to subcellular compartments” [9]. In accordance with these observations, our results showed that all the deranged antioxidant parameters in hypothyroid rats were significantly improved after the concomitant administration of TQ except GPX. Based on all given data, we can assume that the preservation of renal architecture in the hypothyroid+TQ group was achieved in part by the antioxidant capabilities of TQ. In a recent study conducted by Abdel-Wahhab et al., it was reported that GPX did not significantly change in the hypothyroid group treated with eltroxin for one month compared to the untreated hypothyroid group [32]. A more recent study conducted by Farhangi and Tajmiri in 2020 on patients with Hashimoto's thyroiditis treated with powdered N. sativa for 8 weeks. They reported a significant increase in serum antioxidant capacity, SOD, and reduced MDA concentrations with no significant change in the GPX level [33]. These recent studies are supportive to and consistent with these study findings.

Under normal conditions, eNOS is the major source of nitric oxide; however, in renal tissues with pathological lesions, a decoupling process of eNOS is induced, resulting in the generation of O^2-^. Reactive oxygen species are able to induce cellular injury and contribute to fibrosis [34, 35]. In favor with that hypothesis, Masson's trichrome stain revealed extensive collagen deposition in cortical interstitium and in the glomeruli of the hypothyroid group versus few collagen fibers detected in the control group. These results were in line with those of Qiao et al. [35]. In the same context, the current immunohistochemical findings of the hypothyroid group showed strong expression of ASMA in the glomeruli and numerous peritubular ASMA-positive cells supporting the findings of Masson's stain.

The enhancement of oxidative stress as well as the depletion in the endogenous antioxidant defense can initiate a marked immune response [36]. Accordingly, the oxidative stress created by induction of hypothyroidism can explain the interstitial mononuclear cell infiltrate and proliferating fibroblasts observed in the hypothyroid rats. Meanwhile, the present CD68 immunohistochemical study of the same group revealed abundant macrophage infiltrating glomeruli, periglomerular area, and cortical interstitium. On combining TQ with PTU, few collagen fibers could be seen, more or less, similar to the control ones. In addition, attenuation of the CD68+ve cells and mononuclear cell infiltrates as well as retraction of the glomerular ASMA-positive reaction was observed in the TQ-treated group. This could be explained by the well-documented potent anti-inflammatory and the antioxidant promoting effects of TQ which quench the oxidative renal damage induced by thyroxin deficiency [37].

It could be concluded that hypothyroidism-induced renal injury was accompanied with inflammatory changes in the kidneys besides the oxidative damage. The study clearly indicated that TQ might have a potential protective effect against hypothyroidism-induced renal injury, mostly through attenuating the oxidative stress, reversing the redox imbalance, upregulating renal catalase gene expression, and consequently improving inflammatory tissue damage. The increasing levels of thyroid hormones induced by TQ could be a possible mechanism, which indirectly contribute to improving effects induced by TQ on renal function in the present study. The recommended role of TQ supplementation for hypothyroid individuals with high TSH serum levels or high-risk population for developing hypothyroidism needs further investigation.

## Figures and Tables

**Table 1:** Levels of malondialdehyde (MDA), reduced glutathione (GSH), nitric oxide (NO), and catalase gene expression in the studied groups at the end of the experiment.

Variables	Groups			
	Control	TQ	Hypothyroid	Hypothyroid+TQ
MDA	6.6 ± 0.4	2.2 ± 0.3 P1 < 0.001	19.6 ± 0.3 P2 < 0.001	13.6 ± 0.5 P3 < 0.001
GSH	0.41 ± 0.08	0.33 ± 0.05 P1 = 0.09	0.32 ± 0.15 P2 = 0.32	0.53 ± 0.15 P3 = 0.04
NO	26.3 ± 2.2	35.4 ± 10.9 P1 = 0.07	48.6 ± 6.3 P2 < 0.001	59.4 ± 9.2 P3 = 0.03
	
Mean 2^-*Δ*CT^	0.69 ± 0.29	0.89 ± 0.38 P1 = 0.79	0.64 ± 0.21 P1 = 0.94	16.37 ± 2.40 P3 < 0.001
*ΔΔ*Cq expression (fold)	Reference	1.269	0.925	23.36

Data are presented as the mean ± SDM of six rats. Hypothyroid: hypothyroidism; P1: *p* value versus the control group; P2: *p* value versus the control group; P3: *p* value versus the hypothyroid group. Significance is considered at *p* < 0.05.

## Data Availability

The article data will be available upon request.
